# Assisted reproductive technologies, psychosocial stress and low birth weight

**DOI:** 10.1093/emph/eoaa008

**Published:** 2020-04-04

**Authors:** Zaneta M Thayer

**Affiliations:** e1 Department of Anthropology; e2 Ecology, Evolution, Environment & Society Program, Dartmouth College, Hanover, NH, USA

## LOW BIRTH WEIGHT FOLLOWING ASSISTED REPRODUCTIVE TECHNOLOGIES

Assisted reproductive technologies (ARTs), including *in vitro* fertilization (IVF), now account for 1.5% of all births in the USA [1]. Although the majority of children born through these technologies are healthy, ART children are at increased risk of being born with low birth weight (LBW, <2500 g) [1]. Researchers commonly attribute this outcome to parental pre-existing health conditions, medication use and/or specific aspects of ART procedures, including hormone stimulation, media culture conditions and cryropreservation [2]. Given the well-known association between LBW and chronic disease in adulthood *which some evidence suggests is also elevated among ART children *[2], it is important to understand any ‘additional’ factors that could be contributing to the development of LBW following ART.

## EVOLUTIONARY PERSPECTIVES

Organisms have evolved to be sensitive to early environments [2, 3]. A generally reliable indicator of environmental quality is parental wellbeing. Parental stress, which signals environmental adversity, results in developmental adjustments that prioritize accelerated growth and development to ensure survival.

Such adjustments, however, can come at a cost to somatic investment and maintenance. This could explain why maternal stress experience in pregnancy is consistently associated with LBW and chronic disease in offspring [4]. Changes to genomic imprinting patterns may mediate these short- and long-term effects [1, 4].

**Figure 1.**
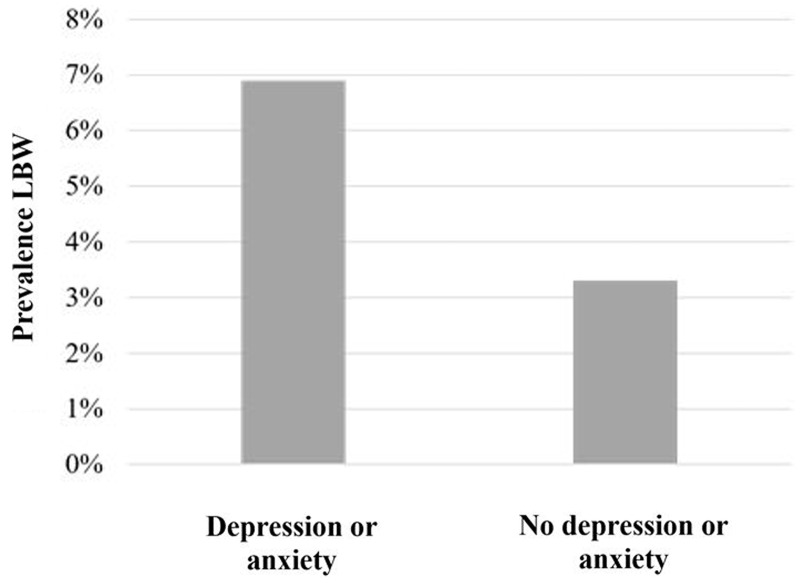
Although data to robustly test the hypothesis that maternal stress contributes to LBW following ART are not readily available, a study [5] among IVF patients found that those who experienced depression or anxiety in the first trimester of pregnancy were twice as likely to have a LBW infant

The ART process, which can be invasive, expensive, and often requires multiple rounds of treatment, is known to be a stressful experience for couples. Unsurprisingly, ART parents have higher self-rated stress, stress hormone levels and depression relative to non-ART parents [5, 6]. Mental health services, however, are currently not offered as standard of care, with a minority of ART couples even being aware of them [6]. ART-associated stress in pregnancy may therefore be an additional and currently underappreciated contributor to LBW within this population (Fig. 1).

## FUTURE IMPLICATIONS

Given the alternative of not conceiving, ART remains a viable option for many individuals and couples experiencing infertility. Insights from our understanding of an evolved, developmental sensitivity to stress suggests that offering mental health services alongside ART treatment could partially reduce LBW associated with these technologies. Additional research is needed to confirm this hypothesis.
